# Comparison between Sugammadex and Neostigmine after Video-Assisted Thoracoscopic Surgery–Thymectomy in Patients with Myasthenia Gravis: A Single-Center Retrospective Exploratory Analysis

**DOI:** 10.3390/jpm13091380

**Published:** 2023-09-15

**Authors:** Hyun-Joung No, Young Chul Yoo, Young Jun Oh, Hye Sun Lee, Soyoung Jeon, Ki Hong Kweon, Na Young Kim

**Affiliations:** 1 Department of Anesthesiology and Pain Medicine, Anesthesia and Pain Research Institute, College of Medicine, Yonsei University, Seoul 03722, Republic of Korea; nhj0410@yuhs.ac (H.-J.N.); seaoyster@yuhs.ac (Y.C.Y.); yjoh@yuhs.ac (Y.J.O.); kihong1@yuhs.ac (K.H.K.); 2 Department of Research Affairs, Biostatistics Collaboration Unit, College of Medicine, Yonsei University, Seoul 03722, Republic of Korea; hslee1@yuhs.ac (H.S.L.); jsy0331@yuhs.ac (S.J.)

**Keywords:** sugammadex, neostigmine, thymectomy, video-assisted thoracoscopic surgery, myasthenia gravis

## Abstract

This single-center retrospective exploratory analysis evaluated the effects of sugammadex compared with neostigmine on postoperative recovery in patients with myasthenia gravis (MG) who underwent video-assisted thoracoscopic surgery (VATS)–thymectomy. This retrospective study included 180 patients with MG, aged >18 years, who received sugammadex (sugammadex group, n = 83) or neostigmine–glycopyrrolate (neostigmine group, n = 88) after VATS–thymectomy between November 2007 and December 2020. Inverse probability of treatment weighting (IPTW) adjustment was performed to balance the baseline characteristics between the two groups. The primary outcome was the length of postoperative hospital stay, and the secondary outcomes were the incidence of postoperative mortality and complications, as well as the postoperative extubation and reintubation rates, in the operating room after VATS–thymectomy; the outcomes were compared between the two groups. After IPTW adjustment, the sugammadex group showed a significantly shorter median postoperative hospital stay than the neostigmine group (4 (2, 4) vs. 5 (3, 6) days, respectively; *p* = 0.003). There were no significant differences between the two groups in the incidences of postoperative complications (including postoperative myasthenic crisis, nerve palsy, atelectasis, and pleural effusion). Patients with MG following VATS–thymectomy who received sugammadex showed a significantly shorter postoperative hospital stay than those who received neostigmine.

## 1. Introduction

Video-assisted thoracoscopic surgery (VATS)–thymectomy is the preferred surgical procedure for patients with myasthenia gravis (MG), and due to the “motionless” lung on the surgical side, it is considered an important indication for one-lung ventilation [1]. Double-lumen endobronchial tubes are the most common method of achieving lung isolation; however, intubation using double-lumen tubes for one-lung ventilation can be difficult compared to the use of single-lumen tubes due to the length, width, and less compliant characteristics of double-lumen tubes [2]. Thus, an adequate neuromuscular blockade is necessary for VATS–thymectomy, even for patients with MG [1,3]. However, general anesthesia in patients with MG carries an increased risk of complications due to sensitivity to neuromuscular blocking agents (NMBAs) [4], and their use can present a vexing dilemma for anesthesiologists. The most concerning issue is the optimal choice and dosage of NMBA and the subsequent reversal agent for patients with MG. 

Sugammadex, a drug which selectively binds to and blocks the action of rocuronium (a widely used non-depolarizing NMBA), has been developed and used in general anesthesia [5,6]. Sugammadex quickly and safely reverses rocuronium-induced neuromuscular blockade and does not interact with acetylcholine receptors or acetylcholinesterase [7]. Various case series have reported the potential effects of sugammadex on the reversal of rocuronium-induced neuromuscular blockade in patients with MG undergoing general anesthesia [8,9,10,11,12,13,14,15,16,17,18]. Mouri et al. were the first to support a potential benefit of sugammadex use over patients who did not receive sugammadex in patients with MG [19]. Additionally, Tsukada et al. reported that postoperative hospital stay was significantly shorter in patients who received a combination of rocuronium and sugammadex than in those in the control group who did not receive any NMBA [20]. 

Therefore, the purpose of this study was to perform a retrospective exploratory analysis in our institution to investigate and to compare the effects of sugammadex and neostigmine on postoperative recovery in patients with MG who underwent VATS–thymectomy. We hypothesized that the use of sugammadex would be associated with a shorter postoperative hospital stay compared with the use of neostigmine combined with glycopyrrolate in patients with MG after VATS–thymectomy. 

## 2. Materials and Methods

### 2.1. Study Population

This single-center retrospective exploratory analysis was conducted after receiving approval from the Institutional Review Board (IRB) and the Hospital Research Ethics Committee of Severance Hospital, Yonsei University Health System, Seoul, Republic of Korea (IRB number, 4-2121-0560; approved on 17 June 2021), and followed the STROBE guidelines for observational studies. The requirement for written informed consent was waived by the IRB owing to the retrospective nature of the anonymous data. The electronic medical records of 180 consecutive patients with MG (aged > 18 years) who underwent VATS–thymectomy with neostigmine or sugammadex between November 2007 and December 2020 were retrieved. 

### 2.2. Intraoperative Management

Anesthesia induction was performed with propofol and remifentanil; after loss of consciousness was confirmed, neuromuscular blockade was induced with rocuronium (Esmeron®, Merck Sharp & Dohme, Seoul, Republic of Korea). Intubation was performed using a left-sided double-lumen endobronchial tube. Radial artery catheterization was performed for continuous pressure monitoring, and 7 Fr central venous catheterization was applied. Anesthesia was maintained with an inhalation anesthetic agent (sevoflurane or desflurane) at a 0.9–1.2 age-adjusted minimal alveolar concentration, combined with a continuous intravenous infusion of remifentanil to target a bispectral index of 40–60 [21,22]. 

After skin preparation and drape was performed, three trocars were inserted. The thymus and mediastinal fat including thymoma were dissected carefully from the inferior thyroid pole to the pericardial fat pad. A specimen was removed, and one 28 Fr thoracic catheter was inserted into the thoracic cavity. After emergence from anesthesia, all patients were transferred to the intensive care unit (ICU) or the post-anesthetic care unit (PACU). The following cases were transferred to the ICU: patients in whom it was determined not to try the extubation, by consensus between the anesthesiologist and the surgeon (these patients were excluded because an NMBA reversal agent was not administered) and patients who were re-intubated in the operation room after extubation due to unstable breathing, or patients in whom although extubation was well-performed, monitoring was needed in case of myasthenia crisis per the surgeon’s decision. The rest of the patients were transferred to the PACU, and then they were moved to the ward after complete recovery.

### 2.3. Neuromuscular Blockade

The monitoring of neuromuscular blockade depth was performed using the acceleromyography-based Train-of-Four (TOF) Watch SX^®^ (Organon Ireland Ltd., Dublin, Ireland) on the adductor pollicis muscle [23]. The neuromuscular blockade was induced by administering rocuronium (Esmeron^®^, Merck Sharp & Dohme, Seoul, Republic of Korea) following the calibration and stabilization of the TOF Watch. Following the attending anesthesiologist’s decision, determined by TOF monitoring, an additional intraoperative NMBA bolus was administered, and neuromuscular blockade was reversed with neostigmine (neostigmine methylsulfate injection, Daihan Pharm. Co., Ltd., Seoul, Republic of Korea) or sugammadex (Bridion^®^, Merck Sharp & Dohme, Seoul, Republic of Korea) after surgery. Additionally, 0.2 mg of glycopyrrolate (glycopyrrolate injection, Reyon Pharm. Co., Ltd. Seoul, Republic of Korea) was used in combination with neostigmine to prevent its muscarinic effects. 

### 2.4. Variables and Outcomes

The demographic and clinical variables included age, sex, body mass index (BMI), American Society of Anesthesiologists (ASA) physical status, comorbidities, and smoking history. To consider the severity of MG, the variables of MG history included disease duration, pyridostigmine administration duration and dose, acetylcholine receptor antibody levels, preoperative quantitative MG score, MG crisis history, and preoperative pulmonary function test findings. Furthermore, we assessed intraoperative variables, such as the duration of anesthesia and operation; blood loss; intraoperative blood transfusion; administered doses of rocuronium, neostigmine, and sugammadex; thymic pathology; and thymoma size. Moreover, postoperative variables included the length of postoperative hospital stay; mortality; incidence of postoperative complications, including postoperative myasthenic crisis, nerve palsy, atelectasis, and pleural effusion; and number of patients extubated in the OR. Postoperative myasthenic crisis was defined as respiratory failure that required prolonged mechanical ventilation (≥3 days) or reintubation within 30 days after thymectomy [24,25]. In addition, perioperative laboratory variables including white blood cell (WBC) count, hematocrit, neutrophil-to-lymphocyte ratio (NLR), and platelet-to-lymphocyte ratio (PLR) were evaluated.

The primary outcome of this study was the length of postoperative hospital stay. The secondary outcomes included the incidences of postoperative mortality and complication, as well as the postoperative extubation and reintubation rates, in the OR after VATS–thymectomy. The primary and secondary outcomes were compared between the sugammadex and neostigmine groups.

### 2.5. Statistical Analyses

Normality for continuous variables was assessed by the Shapiro–Wilk test. Before inverse probability of treatment weighting (IPTW) analysis, continuous variables were presented as the mean ± standard deviation or median (interquartile range) and compared using the independent two-sample *t*-test or Mann–Whiney U test. Categorical variables were presented as the frequency (%) and analyzed using the chi-square or Fisher’s exact test. Due to the retrospective study design, each group had a different number of patients, and the variables were not controlled. IPTW analysis based on the propensity scores was performed to minimize an imbalance of confounding variables between the sugammadex and neostigmine groups [19]. Multiple logistic regression was performed with confounding variables to estimate the propensity scores. Regarding the covariates, basic clinical variables, including age, sex, and BMI, and variables with a standardized difference of >0.2, including ASA physical status, pyridostigmine administration duration and dose, and MG crisis history, were chosen. In the IPTW analysis, the weighted value of the sugammadex group was calculated as p/propensity score, and that of the neostigmine group was calculated as (1–p)/(1–propensity score). Here, p is the probability of allocation in the sugammadex group, and 1–p represents the probability of allocation in the neostigmine group. The allocation ratio is reflected in the numerator of the weighted value [19,26]. The weighted mean and standard deviation of IPTW were calculated by the formulae $\frac{\sum w_{i}x_{i}}{\sum w_{i}}$ and $\frac{\sum w_{i}}{{\left(\sum w_{i}\right)}^{2}-\sum w_{i}^{2}}\sum w_{i}{\left(x_{i}\right.-\left.\bar{x}\right)}^{2},$ respectively, where *w* is the weight and $\bar{x}$ is the weighted mean [27]. We evaluated the balance of confounding factors after weighting according to the standardized differences, calculated by the formula $\frac{{\bar{x}}_{1}+{\bar{x}}_{2}}{\sqrt{\frac{{s_{1}}^{2}+{s_{2}}^{2}}{2}}}$ for continuous variables, where ${\bar{x}}_{1}$ and ${\bar{x}}_{2}$ are means and $s_{1}\;and$ $s_{2}$ are the standard deviations of each group, and $\frac{p_{1}+p_{2}}{\sqrt{\frac{p_{1}\left(1-p_{1}\right)+p_{2}(1-p_{2})}{2}}}$ for categorical variables, where $p_{1}$ and $p_{2}$ are the probabilities of each group. Continuous variables that did not satisfy the normality assumption were analyzed using a weighted Mann–Whitney U test and expressed as medians (interquartile range). The weighted Mann-Whitney U test was calculated using an R package (sjstats) [28]. Confounding factors with a standardized difference of <20% were considered balanced, and a *p*-value of <0.05 was considered statistically significant [29,30]. Repeatedly measured laboratory data were analyzed using a linear mixed model. For variables with statistically significant differences, a post hoc analysis with Bonferroni correction was used to adjust for multiple comparisons. For all statistical analyses, SAS (version 9.4; SAS Institute, Cary, NC, USA) and R (version 4.0.5; R Foundation for Statistical Computing, Vienna, Austria) were the software used.

## 3. Results

**Figure 1:**
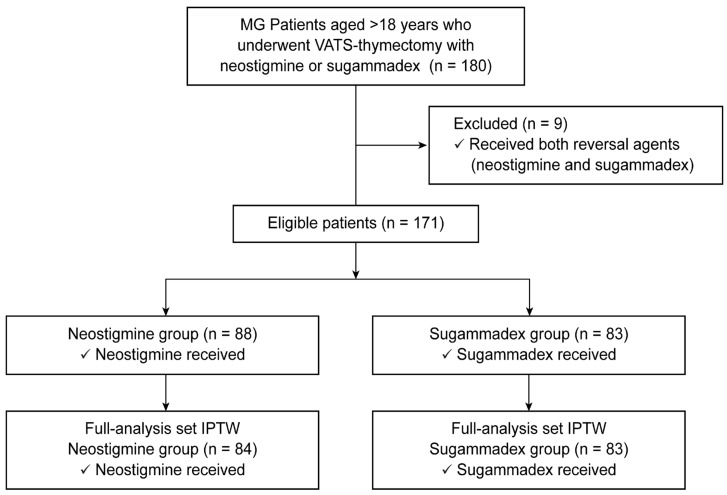
Consolidated Standards of Reporting Trials diagram. MG, myasthenia gravis; VATS, video assisted thoracoscopic surgery; IPTW, inverse probability of treatment weighting.

Among the 180 patients with MG (aged >18 years) who underwent VATS–thymectomy with neostigmine or sugammadex, 9 who received both NMBA reversal agents were excluded. Finally, the remaining 171 patients, deemed eligible for the study, were separated into one of two groups: those administered neostigmine–glycopyrrolate (neostigmine group, n = 88) and those administered sugammadex (sugammadex group, n = 83) (Figure 1). Following IPTW stabilization, the total number of patients in the pseudo dataset was 167, with 84 and 83 in the neostigmine and sugammadex groups, respectively.

**Figure 2:**
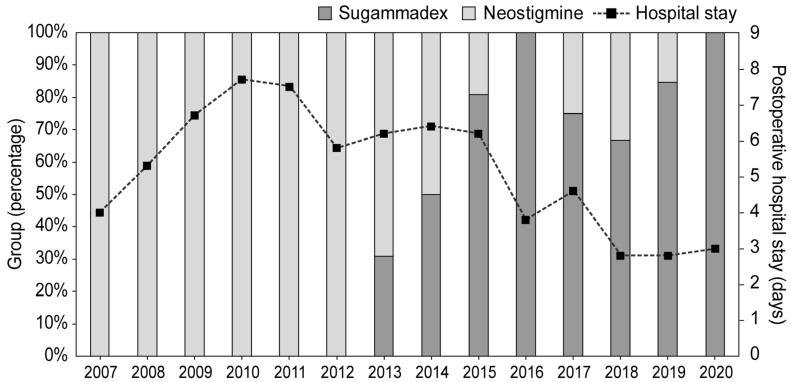
Average length of postoperative hospital stays in days for each year during the study period for all patients.

Figure 2 shows the length of postoperative hospital stays by year throughout the study period for all patients. Before 2013, all patients received neostigmine, whereas after 2013, most patients received sugammadex.

Before applying stabilized IPTW, statistical differences were observed in the ASA physical status and preoperatively administered pyridostigmine dose. After IPTW stabilization, no variable showed any significant between-group differences (Table 1). 

Table 2 shows the operative variables. There were statistical differences in anesthesia and operation time and in dosage of administered rocuronium between the two groups. After the application of IPTW, statistical differences were still seen in anesthesia time and dosage of administered rocuronium. 

The postoperative variables in both groups are shown in Table 3. Patients in the sugammadex group had a significantly shorter median length of hospital stay after VATS–thymectomy than those in the neostigmine group (4 (2, 4) vs. 5 (3, 6) days, respectively; *p* = 0.003), and no deaths were observed in either group. Moreover, the incidences of other postoperative complications, including postoperative myasthenic crisis, nerve palsy, atelectasis, and pleural effusion, were comparable between the two groups (Table 3). Postoperative myasthenic crisis occurred in three patients in the neostigmine group (one patient in the intensive care unit (ICU) on postoperative day (POD) 2 and two in the ward on PODs 4 and 5) and three patients in the sugammadex group (two patients in the ICU on PODs 1 and 9 and one in the ward on POD 2). Additionally, no significant differences were found in the number of patients who were extubated and reintubated in the OR between the two groups.

**Figure 3:**
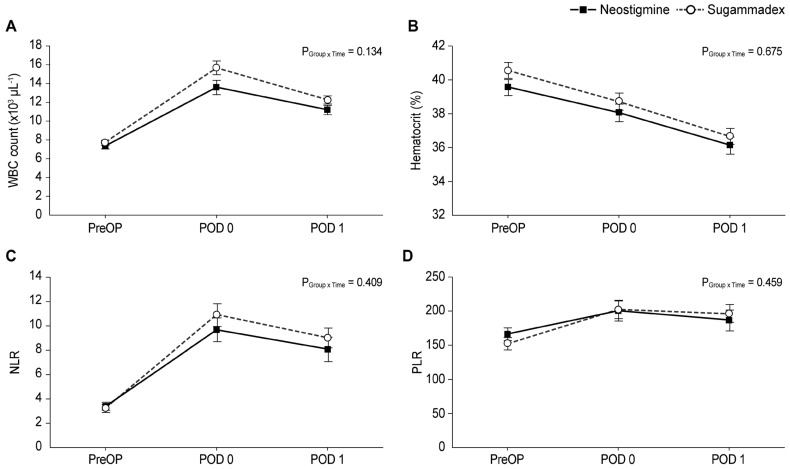
Postoperative laboratory variables; WBC count (**A**), hematocrit (**B**), NLR (**C**), and PLR (**D**). WBC, white blood cell; NLR, neutrophil–lymphocyte ratio; PLR, platelet–lymphocyte ratio; PreOP, preoperation; POD, postoperative day.

Figure 3 shows the perioperative laboratory variables in both groups after IPTW adjustment. No significant between-group differences were observed in WBC count, hematocrit, NLR, or PLR.

## 4. Discussion

This retrospective exploratory analysis demonstrated that patients who received sugammadex had significantly shorter postoperative hospital stays than those who received neostigmine. 

Neuromuscular blockade during general anesthesia was previously avoided in patients with MG, owing to the high sensitivity of the muscles to NMBAs [31,32]. However, several case reports have demonstrated the use of rocuronium and sugammadex in patients with MG [1,8,9,10,12,13,14,15,16,17,18,33,34,35,36,37]. Patients in different stages of MG who underwent various surgical interventions—including thymectomy—showed rapid reversal of rocuronium-induced muscle relaxation after sugammadex administration, without significant occurrence of postoperative complications [1,8,9,10,12,14,15,16,17,18,33,36]. A few cases have been reported in which symptoms were alleviated after AChEI administration in patients with incomplete recovery following sugammadex administration and accompanying muscle weakness [13,34,35,37]. However, the comparative effects of the use of sugammadex and neostigmine on perioperative outcomes in patients with MG following thymectomy remain unexplored.

In this study, patients in the sugammadex group had a significantly shorter postoperative hospital stay than those in the neostigmine group following VATS–thymectomy, which is consistent with other studies [19,20]. Mouri et al. demonstrated that compared with the patients in the control group who did not receive sugammadex, those who received rocuronium–sugammadex had a significantly lower incidence of postoperative myasthenic crisis, as well as a significant reduction in the median total hospital costs and median postoperative hospital stay [19]. Tsukada et al. reported no in-hospital deaths in patients administered rocuronium and sugammadex and no difference in the use of plasma exchange and immunoglobulins following thymectomy compared with those in the control group, who did not receive any NMBA. Additionally, postoperative hospital stays were significantly shorter in patients who received rocuronium–sugammadex than in those in the control group [20]. Nevertheless, there are some differences between the findings in these previous studies and those in this study. In the previous studies, patients in the control group either did not receive an NMBA [20] or were not treated with the specific reversal agents used in the control group [19]. In contrast, patients in the control group of this study received neostigmine and glycopyrrolate, which is a strength of this work. 

Additionally, all patients in this study underwent VATS–thymectomy, whereas 65.9–72.3% of patients in the previous studies underwent open thoracotomy thymectomy and/or transsternal thymectomy [19,20]. Considering the rapid increase in the clinical use of VATS–thymectomy, accompanied by the advancements and popularity of VATS technology [38], this study offered new evidence on the potential benefits of rocuronium and sugammadex in shortening the postoperative hospital stay after thymectomy in patients with MG. 

Regarding the secondary outcomes, there was no case of in-hospital mortality in either group. Furthermore, postoperative respiratory failure attributed to myasthenic crisis occurred in three patients in the neostigmine group and three in the sugammadex group. Myasthenic crisis should be distinguished from cholinergic crisis, which is generally caused by an excessive use of cholinesterase inhibitors [39]. However, because pyridostigmine was maintained in all of these patients, the respiratory failure was considered to be a result of postoperative myasthenic crisis. Subsequently, all patients recovered after reintubation and receiving ventilator-related care. In this study, the incidence rates of complications corresponding to secondary endpoints were comparable between the two groups. Larger studies are required to validate these findings. 

Due to the insufficient number of patients with MG undergoing VATS–thymectomy, this study included all patients from November 2007, when VATS–thymectomy was actively initiated in this institution, to December 2020. The improvement in surgical techniques over the long study period may have influenced the outcome; thus, we depicted the average length of postoperative hospital stay in days for each year during the study period in Figure 2. Between 2007 and 2012, only neostigmine was used because sugammadex has only been administered since 2013. Because the average lengths of postoperative hospital stay between 2012 and 2015 were similar, it is unclear whether the surgical technique affected the postoperative hospitalization period. Additionally, when comparing the lengths of postoperative hospital stay between 2015, when neostigmine and sugammadex were used, and 2016, when only sugammadex was used, it is unclear whether the reduced length of postoperative hospital stay in 2016 was due to the improvement of surgical techniques over 1 year. To exclude the influence of surgical techniques, only surgeries since 2013, when sugammadex was available, should have been included. However, while sugammadex usage was too high since 2013, neostigmine usage was too high before 2013. Therefore, patients were included over a long study period, and different numbers of patients were included for comparison between the two groups. There were a few standardization issues, such as the anesthetic agents used (sevoflurane or desflurane), although this issue does not directly affect the outcomes. Moreover, several factors may affect LOS and mortality rates, and reversal of neuromuscular blockade is just one of those factors, particularly when including patients over a 13-year period; although IPTW analysis was performed to minimize such imbalances, these can be considered major limitations of this study. In optimizing the treatment for these patients in a single institution, even a longitudinal effect (learning effect) can lead to bias, which can affect the length of hospital stay. Thus, further prospective controlled trials are needed to add clinical significance to the existing literature. 

It is noteworthy that the sugammadex group presented with a longer duration of anesthesia and longer operative time, as it should be expectable to improve the surgical time by providing better relaxation and a better surgical field, and improved potential reversal effects could help also shorten the extubation time. This is because the amounts of NMBA administered to the two groups have not been adjusted, which is another important limitation of the current study. The neuromuscular blockade depth was monitored by the attending anesthesiologists during surgery, using the acceleromyography-based TOF Watch SX, to determine the need for additional NMBA administration. However, there were no records of the degree of neuromuscular blockade depth, and there were only records of the total administered dosage of rocuronium. Thus, the retrospective nature of this study is its main drawback, which makes the data vulnerable to bias and confounding factors. In a future prospective study, an accurate comparison regarding this needs to be made between the sugammadex group applying the deep neuromuscular blockade and neostigmine group applying the moderate neuromuscular blockade.

Another limitation is the “one size fits all” approach to administering neostigmine. While different doses of sugammadex were administered to the patients in the sugammadex group, all patients in the neostigmine group received 1 mg of neostigmine, combined with 0.2 mg of glycopyrrolate. Thus, future studies should compare the effects of different doses of neostigmine.

## 5. Conclusions

To conclude this retrospective exploratory analysis, patients who received sugammadex had significantly shorter postoperative hospital stays than those who received neostigmine, and sugammadex use may help improve anesthetic management strategies in patients with MG undergoing VATS–thymectomy. However, it is important to keep in mind that there are several factors affecting the length of hospital stay and mortality, and that the reversal agent of the neuromuscular blockade is only one of those factors, especially in long-term follow-up studies. Thus, further large-scale prospective trials are required in a different setting to establish definite evidence.

## Figures and Tables

**Table 1:** Demographic characteristics after using inverse probability of treatment weighting.

Variables	Original Dataset before IPTW				Pseudo Dataset after IPTW			
	Neostigmine (n = 88)	Sugammadex (n = 83)	*p*-Value	SMD	Neostigmine (n = 84)	Sugammadex (n = 83)	*p*-Value	SMD
Age, year	44 (30, 53)	44 (30, 54)	0.733	0.037	40 (28, 52)	46 (31, 54)	0.427	0.143
Sex, female	59 (67)	57 (69)	0.820	0.035	53 (64)	54 (65)	0.904	0.021
Body mass index, kg/m^2^	22.4 (20.6, 25.6)	22.5 (21.0, 24.7)	0.908	0.092	22.4 (20.4, 25.5)	23.0 (21.0, 24.7)	0.773	0.049
ASA physical status			<0.001 *	0.714			0.676	0.072
II	69 (78)	38 (46)			55 (66)	52 (62)		
III	19 (22)	45 (54)			29 (34)	31 (38)		
Comorbidities								
Hypertension	14 (16)	13 (16)	0.965	0.007	13 (15)	13 (16)	0.952	0.010
Diabetes	6 (7)	3 (4)	0.498	0.144	5 (6)	3 (3)	0.425	0.122
Smoking history	19 (22)	17 (20)	0.859	0.027	17 (21)	21 (25)	0.553	0.101
Smoking history, PYRs	0 (0, 0)	0 (0, 0)	0.750	0.166	0 (0, 0)	0 (0, 0)	0.599	0.001
Preoperative MG history								
Disease duration, months	6 (2.75, 24)	8 (3, 27)	0.492	0.133	7 (2.5, 24)	7 (3, 17)	0.787	0.024
Pyridostigmine duration, months	2 (1, 9.5)	4 (1, 21)	0.118	0.296	2 (1, 13)	3 (1, 10)	0.873	0.090
Pyridostigmine dose, mg/day	240 (180, 480)	240 (180, 360)	0.020 *	0.294	240 (180, 480)	240 (180, 360)	0.605	0.001
Ach receptor antibody, nmol/L	10.4 ± 4.8	10.2 ± 5.6	0.799	0.040	10.5 ± 4.6	10.1 ± 5.5	0.656	0.074
Preoperative QMG score	9 (6, 14)	9.5 (5, 13)	0.514	0.049	9 (5, 13)	9 (5, 13)	0.687	0.020
MG crisis history	1 (1)	6 (7)	0.058	0.308	2 (2)	3 (4)	0.492	0.121
Preoperative pulmonary function test								
FEV_1_, L	2.6 ± 0.7	2.7 ± 0.7	0.312	0.161	2.7 ± 0.7	2.6 ± 0.7	0.637	0.084
FVC, L	3.0 (2.6, 3.6)	3.2 (2.8, 3.8)	0.100	0.288	3.1 (2.6, 3.8)	3.3 (2.7, 3.7)	0.575	0.066

Values are the mean ± standard deviation, median (interquartile range), or number (%) of patients. * *p* < 0.05. SMD, standardized mean difference; ASA, American Society of Anesthesiologists; PYR, pack-year; Ach, acetylcholine; QMG score, quantitative myasthenia gravis score; MG, myasthenia gravis; FEV_1_, forced expiratory volume in 1 s; FVC, forced vital capacity; IPTW, inverse probability of treatment weighting. Counts in the weighted data may not sum to the expected totals owing to rounding. The percentages may not total 100 because of rounding, and disagreements between numbers and percentages in the weighted data are the result of rounding of the noninteger numerical values.

**Table 2:** Operative variables.

Variables	Original Dataset before IPTW			Pseudo Dataset after IPTW		
	Neostigmine (n = 88)	Sugammadex (n = 83)	*p*-Value	Neostigmine (n = 84)	Sugammadex (n = 83)	*p*-Value
Intraoperative variables						
Anesthesia time, min	155 (135, 190)	190 (140, 225)	0.005 *	155 (135, 190)	190 (140, 230)	0.012 *
Operation time, min	105 (84.5, 138)	133 (87, 165)	0.018 *	104 (84, 137)	124 (81, 165)	0.085
Blood loss, ml	0 (0, 50)	0 (0, 20)	0.113	0 (0, 50)	0 (0, 20)	0.400
Intraoperative RBC transfusion	0	0	1.000	0	0	1.000
Administered rocuronium, mg	50 (27.5, 50)	50 (50, 50)	0.002 *	50 (25, 50)	50 (50, 50)	0.006 *
Administered neostigmine, mg	1 (1, 1)	-	-	1 (1, 1)	-	-
Administered sugammadex, mg	-	200 (200, 200)	-	-	200 (200, 200)	-
Thymic pathology			0.372			0.231
Normal thymus	5 (6)	2 (2)		5 (6)	1 (1)	
Thymoma	40 (45)	48 (58)		39 (46)	49 (59)	
Thymic/Follicular hyperplasia	36 (41)	28 (34)		34 (40)	26 (31)	
Others	7 (8)	5 (6)		7 (8)	7 (8)	
Thymoma size, cm	3.6 (2.5, 5)	4 (2.95, 5.45)	0.254	3.7 (2.6, 5)	4 (2.9, 5.5)	0.380

Values are the median (interquartile range) or number (%) of patients. * *p* < 0.05. RBC, red blood cell. IPTW, inverse probability of treatment weighting. Counts in the weighted data may not sum to the expected totals owing to rounding. The percentages may not total 100 because of rounding, and disagreements between numbers and percentages in the weighted data are the result of rounding of the noninteger numerical values.

**Table 3:** Postoperative variables.

Variables	Original Dataset before IPTW			Pseudo Dataset after IPTW		
	Neostigmine (n = 88)	Sugammadex (n = 83)	*p*-Value	Neostigmine (n = 84)	Sugammadex (n = 83)	*p*-Value
Length of postoperative hospital stay, days	5 (3, 7)	4 (2, 5)	0.001 *	5 (3, 6)	4 (2, 4)	0.003 *
Mortality	0	0	1.000	0	0	1.000
Postoperative complication						
Postoperative myasthenic crisis	3 (3)	3 (4)	1.000	2 (2)	2 (3)	0.908
Nerve palsy	1 (1)	0 (0)	1.000	1 (1)	0 (0)	1.000
Atelectasis	0 (0)	1 (1)	0.485	0 (0)	1 (1)	1.000
Pleural effusion	1 (1)	0 (0)	1.000	1 (1)	0 (0)	1.000
Extubated patients in the OR	84 (95)	81 (98)	0.683	80 (96)	82 (99)	0.176
Reintubated patients in the OR	4 (5)	1 (1)	0.369	3 (3)	1 (2)	0.533
Finally extubated patients in the OR	80 (91)	80 (96)	0.145	78 (93)	81 (97)	0.201

Values are the median (interquartile range) or number (%) of patients. * *p* < 0.05*. OR, operating room; IPTW, inverse probability of treatment weighting.

## Data Availability

The original contributions presented in the study are included in the article. Further inquiries can be directed to the corresponding authors.
